# Research progress on the allergic mechanism, molecular properties, and immune cross-reactivity of the egg allergen Gal d 5

**DOI:** 10.3389/fnut.2023.1205671

**Published:** 2023-06-07

**Authors:** Wei Jiaqi, Cong Yanjun

**Affiliations:** Beijing Advanced Innovation Center for Food Nutrition and Human Health, College of Food and Health, Beijing Technology and Business University, Beijing, China

**Keywords:** egg allergen, α-livetin, epitope, immune cross-reactivity, prospects

## Abstract

Eggs and their products are commonly consumed in food products worldwide, and in addition to dietary consumption, egg components are widely used in the food industry for their antimicrobial, cooking, and other functional properties. Globally, eggs are the second most common allergenic food after milk. However, current research on egg allergy primarily focuses on egg white allergens, while research on egg yolk allergens is not comprehensive enough. Therefore, summarizing and analyzing the important allergen α-livetin in egg yolk is significant in elucidating the mechanism of egg allergy and exploring effective desensitization methods. This paper discusses the incidence, underlying mechanism, and clinical symptoms of egg allergy. This article provides a comprehensive summary and analysis of the current research status concerning the molecular structural properties, epitopes, and immune cross-reactivity of the egg yolk allergen, Gal d 5. Additionally, it examines the effects of various processing methods on egg allergens. The article also offers suggestions and outlines potential future research directions and ideas in this field.

## 1. Relationship between egg allergy and food allergy

Food allergy, also known as food hypersensitivity, refers to an abnormal or excessive immune response caused by food allergens, medically referred to as an allergic reaction to food. In the past few decades, the number of patients with food allergies and the incidence of food allergies has been gradually rising. The prevalence of food-induced asthma and allergic rhinitis has increased significantly, and it is now considered a serious public health problem in developed countries. In the United States, it affects about 8% of children (corresponding to >5.6 million United States children), 10.8% of adults (corresponding to >26 million United States adults) (1, 2). In other parts of the world, such as Vietnam, South Africa, the incidence of food allergies is gradually increasing, with urban areas being affected more severely than rural areas (2–5).

In theory, any food protein can be considered a potential allergen. However, the most common allergenic foods include milk, eggs, wheat, soy, peanuts, tree nuts, fish, and seafood (6–8). Most food-induced allergic reactions are type I hypersensitivity reactions, characterized by immediate hypersensitivity responses triggered by cross-linking immunoglobulin E (IgE) by allergens. This interaction induces the production of mediators, such as histamine and bradykinin. Food allergies can lead to skin, respiratory, gastrointestinal, and cardiovascular disorders, presenting typical symptoms like urticaria, nausea, abdominal pain, vomiting, respiratory distress, and hypotension. In some cases, these reactions can even be life-threatening (9).

Eggs and their derivative products are commonly consumed worldwide (10). Due to their high protein content and other properties, consuming a certain number of eggs every day can benefit health (10, 11). In addition to dietary consumption, egg components are also widely used in the food industry for their antimicrobial, cooking, and other functional properties (12). Some nutritional and medicinal preparations also contain egg protein (13). Eggs are now an important part of the daily diet. However, the increasing egg consumption has led to the emergence of various nutritional and health issues. Globally, egg allergy is the second most common food allergy after milk allergy. The prevalence of egg allergy in Greece is 0.07%, while in Germany and the United Kingdom, it is >2% (14). The worldwide prevalence of egg allergy is about 2.5% in adults and 6–8% in children, and these rates are increasing each year (15–17). The HealthNuts study in 2011 found that 8.9% of children in Australia were allergic to eggs (18, 19). Currently, there is no systematic epidemiological survey on egg allergy in China. However, in 2012, some clinical reports indicated that about 4% of children aged 0–2 years in Chongqing, Zhuhai, and Hangzhou were allergic to eggs (20), indicating that, even in China, egg allergy is a very common food allergy. It not only affects the quality of life of patients allergic to egg but also imposes a heavy economic burden on the country and the government.

The incidence rate of egg allergy is higher in infants and young children, in a recent evaluation of anaphylaxis in infants younger than 12 months of age, eggs were found to be the most common food trigger (21). However, past studies have found that anaphylaxis triggered by eggs can be life-threatening in children with asthma (22). Allergic reactions to eggs in infants and young children typically manifest as urticaria, vomiting, and angioedema. Such reactions may be accompanied by symptoms of respiratory and cardiovascular disorders, including coughing, wheezing, chest and throat tightness, hypotension, and collapse. However, egg allergy exhibits various differences compared to other common childhood food allergies, such as milk and peanut. Both egg and milk allergens are heat-labile and possess poor digestive stability, whereas peanut allergens are heat-stable and demonstrate greater digestive stability. In addition, egg allergy is associated with a lower risk of fatal reactions (7%) compared to peanut and milk allergies (17 and 48%, respectively). Moreover, compared to peanut allergy, egg and milk allergies usually resolve in childhood, while peanut allergy usually persists into adulthood. About two-thirds of children allergic to eggs remain no longer allergic by the age of 5, and 85–90% of children allergic to milk can tolerate it by the age of 3. In contrast, only 20% of children with peanut allergy develop tolerance by the age of 6 (23).

## 2. Mechanism of egg allergy mediated by mitogen-activated protein kinase

**Figure 1:**
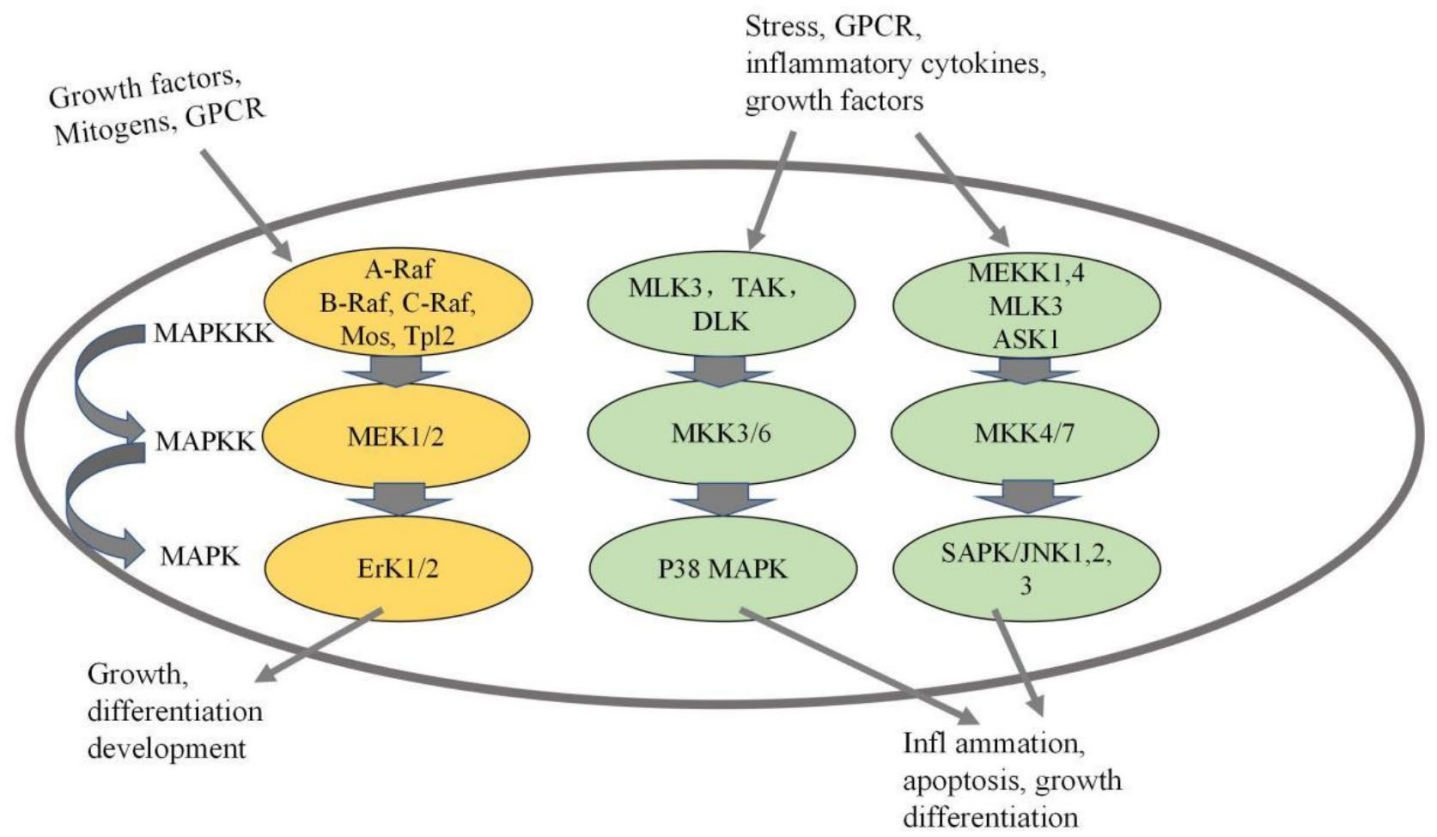
MAPK pathways mediated by ERK, JNK, and p38 protein kinases.

Lianto et al. (24) found that quail eggs, especially quail egg protein, play an important role in regulating protease-activated receptor (PAR)-2-mediated mitogen-activated protein kinase and NF-kB translocation. Wang et al. (25) demonstrated that egg allergens regulate asthma-related genes through the MAPK/JNK and MAPK/p38 pathways. We speculate that allergic mechanism of Gal d 5 may mediated by MAPK signaling pathway, which will become the focus of research. The MAPK signaling pathway has gained recognition as an essential mechanism involved in allergic reactions (26). MAPK serves as a critical growth signal-regulating protein that receives receptor signals and transmits them to the cell nucleus (27). The MAPK signaling pathway plays a pivotal role in various cellular processes, including cell proliferation, differentiation, apoptosis, inflammation, and immune response (28). Numerous stimuli, such as mechanical stimulation, cytokines, growth factors, hormones, and cellular stress, can activate the MAPK signaling pathway (29). Activation of the entire pathway is facilitated by a series of protein kinases (MAPKKK-MAPKK-MAPK). Mammals express at least four distinct MAPK pathways, including p38MAPK, ERK, JNK, and ERK5 pathways, with the first three being more extensively understood (see Figure 1) (30). The mechanism of Gal d 5 needs to be studied in depth.

## 3. Current research status on egg allergen α-livetin

Egg allergens are concentrated in egg white and egg yolk. Of them, egg white allergens have been more extensively studied. Four egg white proteins have been identified as egg allergens. These four proteins are ovomucoid (OVM), which is named Gal d 1 according to the naming convention of the Allergen Nomenclature Sub-Committee of the World Health Organization/International Union of Immunological Societies (WHO/IUIS); ovalbumin (OVA), named Gal d 2; ovotransferrin (OVT), named Gal d 3; and lysozyme (Lys), named Gal d 4 (31). However, the oral food challenge (OFC) has demonstrated that 9.1% of egg-allergic children also show positive reactions to heated egg yolk containing a small amount of egg white (32). Increasing evidence suggests that egg allergies are mediated by both egg white allergens and egg yolk allergens. Recent studies have confirmed that many children diagnosed with egg allergies are actually allergic to egg yolk (33–35). Egg yolk allergy (EYA) has garnered increasing attention in recent years. The primary allergens identified in egg yolk are α-livetin (also known as chicken serum albumin), designated as Gal d 5, and yolk glycoprotein42 (YGP42), referred to as Gal d 6 (36, 37). Currently, there is limited research on egg yolk allergens due to the lower incidence of EYA compared to egg white allergy and the inefficient methods for isolating and purifying egg yolk allergens. Therefore, the analysis and diagnosis of egg yolk allergens can improve the diagnosis of egg allergy, and studying egg yolk allergens plays an essential role in egg allergy research.

α-livetin was the first allergen discovered in egg yolk. It is a water-soluble 69-kDa globular glycoprotein, accounting for approximately 7% of egg yolk proteins. Huang et al. (38) analyzed Gal d 5 using the Expasy ProtParam server. This protein consists of 615 amino acids, with an isoelectric point of 5.51, a chemical formula of C_3060_H_4818_N_826_O_936_S_55_, an instability index of 45.12, and a grand average hydropathicity (GRAVY) of −0.347. The secondary structure of the protein was predicted using Garnier-Robson and Cho-Fasman methods, and the secondary structure of Gal d 5 was analyzed using SOPMA and DNAstar software. Their analysis revealed that α-helices, extended chains, β-sheets, and irregular curls accounted for 66.18, 4.23, 2.93, and 26.67% of the Gal d 5 sary structure, respectively.

## 4. Current research status on epitopes of the egg allergen Gal d 5

Epitopes serve as the foundation for allergy research. In food allergic reactions, allergenic proteins directly interact with antibodies through allergenic epitopes (39). Therefore, investigating the linear and conformational epitopes of egg allergens is crucial for developing hypoallergenic egg products and vaccines targeting egg allergies. Epitopes, also known as antigenic determinants, refer to specific chemical groups within antigen molecules that determine antigen specificity. They are the basic structural units that T cell receptors (TCR), B cell receptors (BCR), or antibody-specific binding. Epitopes typically comprise 5 to 17 amino acid residues or 5 to 7 polysaccharide residues or nucleotides (40). They are usually classified into linear and conformational epitopes based on their structural properties–the former comprises contiguous amino acids, and the latter comprises non-contiguous amino acid residues folded into a spatial structure. Epitopes can also be classified into T-cell and B-cell epitopes based on the cells they bind to (41). Linear epitopes can bind to both T-cells and B-cells; however, conformational epitopes only bind to B-cells.

At present, the understanding of food allergen epitopes remains in its early stages; however, relevant data on milk, peanut, and egg allergen epitopes have been obtained. The findings from later-stage research are anticipated to be applied in medical clinical diagnosis and treatment, playing a crucial role in the food industry (42, 43). Common techniques for identifying food allergen epitopes encompass experimental methods, such as enzymatic hydrolysis, X-ray diffraction, peptide libraries, and peptide scanning technologies, as well as prediction methods based on bioinformatics. While experimental methods offer numerous advantages, they also possess some drawbacks, including poor specificity, time-consuming processes, and high expenses associated with enzyme hydrolysis and peptide scanning. The bioinformatics-based prediction is a new epitope localization strategy. Epitope prediction facilitates the discovery of new epitopes (44) and plays a significant role in epitope-based food allergy research, providing clear desensitization targets for developing hypoallergenic or non-allergenic foods.

Research on epitopes of egg white allergens is far more extensive than that on epitopes of egg yolk allergens. For example, as early as 1992, the key B-cell epitopes of ovalbumin (OVA) were determined to be between positions 41–172 and 301–385 through peptide membrane and dot blot assays (45). Later, the major B-cell epitopes of OVA were found to be located at positions 38–49, 95–102, 191–200, 243–248, and 251–260 in its complete amino acid sequence. Its major sensitizing amino acids were primarily hydrophobic. The structural region with β-sheet or β-turn is formed by partially polar and charged amino acid residues, where the amino acids at positions 95–102 form a separate α-helical structure (46). The amino acids at positions 323–330 were identified as T-cell epitopes of OVA (47). Recently, Jankovicova used magnetic beads with biochemical functionality to locate the epitopes of OVA through microfluidic channels and found that the residue HIATNAVLFFGR (positions 371–382) in OVA is its major B-cell epitope. This epitope was also found to hold great potential to be used as a vaccine for egg allergy (48).

**Figure 2:**
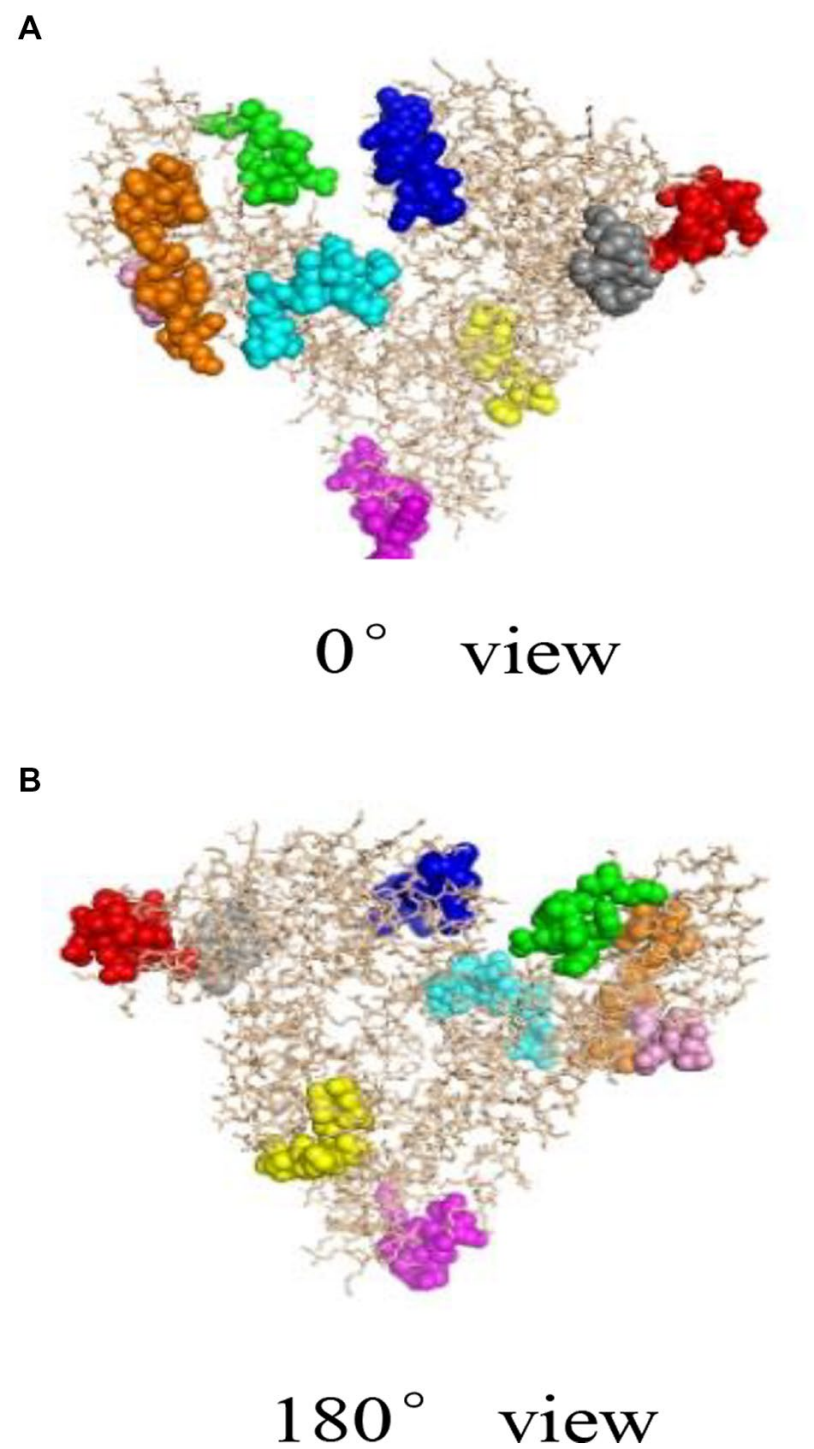
The linear epitope region of α-livetin (49). Red represents the linear epitope of amino acid (aa) 81–92, gray represents the linear epitope of aa118–125, blue represents the linear epitope of aa137–148, yellow represents the linear epitope of aa250–257, purple represents the linear epitope of aa390–400, cyan represents the linear epitope of aa490–500, orange represents the linear epitope of aa522–535, green represents the linear epitope of aa542–551, and pink represents the linear epitope of aa565–570. **(A)** represents the conformational epitope region of a-livetin at 0° view, **(B)** represents the conformational epitope region of a-livetin at 180° view.

**Figure 3:**
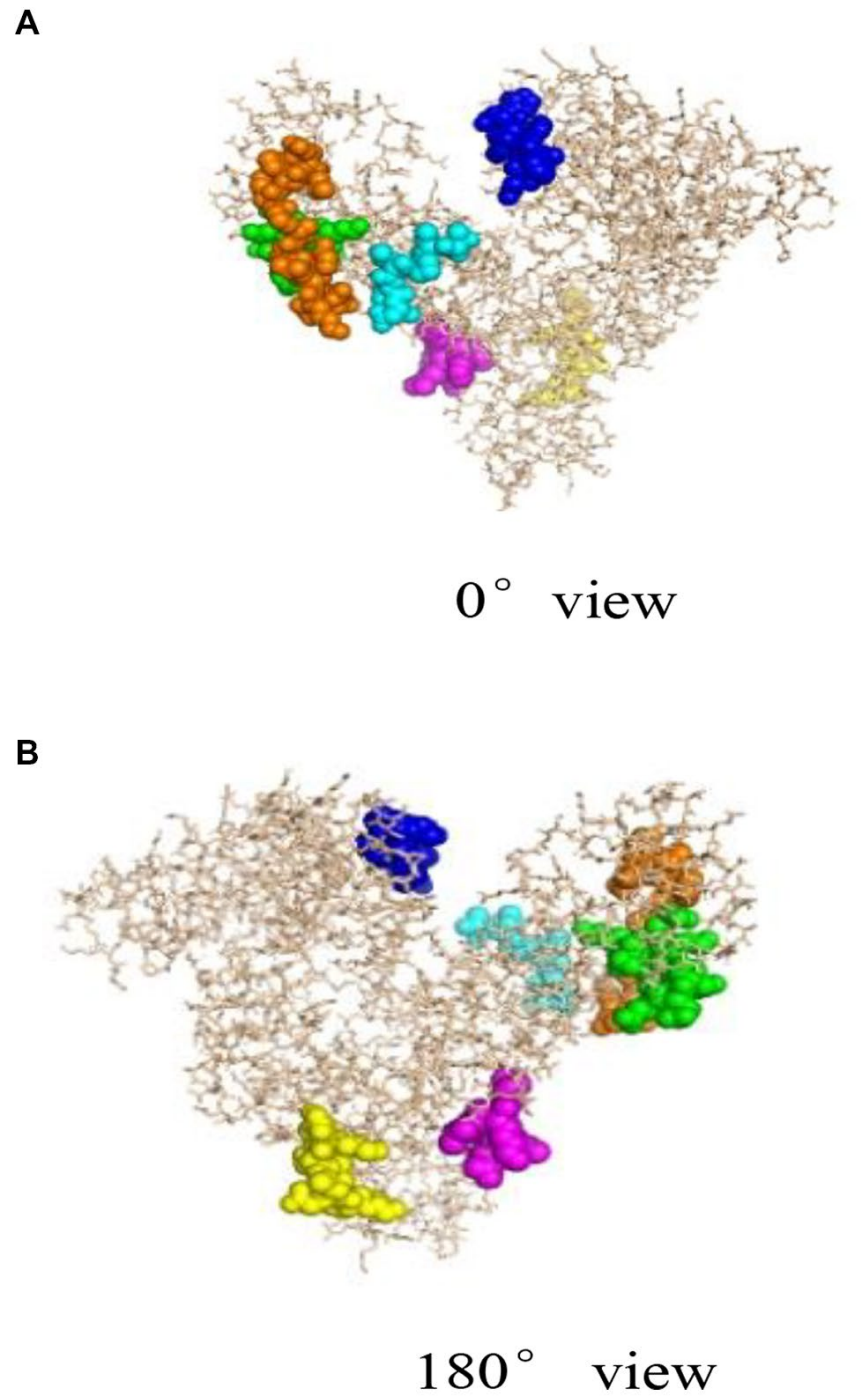
Spatial conformation and conformational epitope region of α-livetin (49). Blue represents the conformational epitope of aa138–146, yellow represents the conformational epitope of aa325–332, purple represents the conformational epitope of aa406–411, cyan represents the conformational epitope of aa494–499, orange represents the conformational epitope of aa521–534, and green represents the conformational epitope of aa561–571. **(A)** represents the linear epitope region of a-livetin at 0° view, **(B)** represents the linear epitope region of a-livetin at 180° view.

Bioinformatics plays an important role in revealing the structural information of Gal d 5. Our research team, led by Wang Guangyu, searched for a homologous model of the egg allergen based on the SWISS-MODEL homology modeling results (49). Wang Guangyu selected the recombinant model comprising human serum albumin and palmitic acid, with SMTL ID 4bke.1.A, as a template for linear epitope identification. The sequence similarity between this model and α-livetin was 47.7%, with an amino acid sequence coverage of AA30–612. Prediction analysis using Bepipred 1.0 revealed nine linear epitope regions in the α-livetin sequence. These regions were labeled using the PyMoL software (Figure 2; colored regions). The positions of these nine epitope regions in the amino acid sequence were 81–92 (red), 118–125 (gray), 137–148 (blue), 250–257 (yellow), 390–400 (purple), 490–500 (cyan), 522–535 (orange), 542–551 (green), and 565–570 (pink) (Figure 2). Moreover, Wang Guangyu predicted six conformational epitope regions in α-livetin using the DiscoTope network database at positions 138–146 (blue), 325–332 (yellow), 406–411 (purple), 494–499 (cyan), 521–534 (orange), and 561–571 (green) in the amino acid sequence (Figure 3; colored areas).

In addition to predicting linear and conformational epitopes, Huang et al. (38) used the TMHMM server^1^ to analyze Gal d 5. They found that the protein contained transmembrane regions and that the predicted number of transmembrane regions in Gal d 5 is greater than 1, indicating its presence in the extracellular matrix. The B-cell epitopes of Gal d 5 were predicted using the ABCpred, BepiPred, BCPREDS, and SVMTriP methods and verified using immunoblotting and cellular immunology. In addition, HLA-DRB1 × 0901, DRB1 × 1,501, and DRB1 × 701 were chosen as reference bases for predicting Gal d 5, and the IEDB online server was used to predict the T-cell epitopes of Gal d 5. As shown in Table 1, six B-cell epitopes (AA118–125, 137–148, 390–400, 430–444, 454–463, 490–496) and seven T-cell epitopes (AA52–57, 126–140, 163–170, 177–185, 242–247, 433–441, 599–613) were predicted in Gal d 5.

**Table 1:** Prediction of the B-cell and T-cell dominant epitopes of Gal d 5 (50).

	Method	Location	Sequence
B-cell epitopes	ABCpred, BepiPred, BCPREDS, SVMTriP	118–125	CSKADPER
		137–148	PDFVQPYQRPAS
		390–400	KTDNPAECYAN
		430–444	DFLKSILIRYTKKMP
		454–463	TGKKMTTIGT
		490–496	RKQETTP
T-cell epitopes	IEDB	52–57	ITFAQY
		126–140	KADPERNECFLSFKVSQPDFV
		163–170	LGHFIYSV
		177–185	LYAPAILSF
		242–247	RQLIYL
		433–441	SILIRYTK
		599–613	EGANLIVQSRATLGI

## 5. Current research status on Gal d 5 cross-reactivity

Food cross-reactivity allergy can occur when a person consumes two or more different food products, they are allergic to (51, 52). Some studies have shown that cross-reactivity to completely different allergens can occur in the same organism. This cross-reactivity is attributed to two allergens having similar antigenic epitopes. For example, patients allergic to spores might exhibit cross-reactivity to alternative foods (50). Previous studies have proved cross-reactivity between apple and celery, cashews and walnuts, and bananas and pigweed (53–55), and cross-reactivity allergy between other foods has also been reported (56). Currently, IgE antigenicity and cross-reactivity cannot be reliably used as indicators of the presence or likelihood of occurrence of food allergy (57). Similarly, the structural properties of allergens do not always correlate with the severity of allergenicity or allergic reactions (58).

According to the evaluation strategy recommended by FAO/WHO (2001), the criteria for determining cross-reactivity between a test protein and a known allergen are >35% homology or at least six consecutive identical amino acids. However, relying solely on the criterion of six consecutive identical amino acids can easily result in false positive outcomes due to the random arrangement and combination of amino acids in natural protein sequences. Consequently, in 2003, Codex Alimentarius Commission (CAC) revised these criteria to consider potential cross-allergenicity if any 80 amino acid sequences exhibit more than 35% homology or if eight consecutive amino acids are identical. Nevertheless, amino acid sequence homology should not be the only criterion for determining the potential allergenicity of proteins. Therefore, combining experimental testing with clinical diagnosis is an effective method for confirming the existence of cross-reactivity.

Presently, research on the cross-reactivity of egg allergens is ongoing. Moghtaderi et al. conducted skin prick tests on 52 egg-allergic children using fresh extracts of pigeon, duck, goose, turkey, and quail eggs, finding that 50 (96.1%) of the children exhibited allergic reactions to at least one type of egg (59). Mitomori et al. demonstrated a clinical cross-reaction between eggs and quail eggs (60). Hemmer et al. identified cross-allergic reactions to eggs in individuals allergic to poultry feathers, known as bird-egg syndrome (61). Additionally, simultaneous reactions with bird feathers and egg yolk are often observed in allergy tests, both *in vivo* and *in vitro*, and strong mutual cross-inhibition is noted between bird feather extracts and egg yolk but not between feather extracts and egg white (36). In bird-egg syndrome, the main sensitization is caused by inhalation of airborne bird allergens with cross-reactivity to α-livetin (Gal d 5) in egg yolk.

Wang Guangyu (49) of our research group used the Uniprot method to analyze potential cross-reactivity between eggs and milk. It was found that the sequence similarity between α-livetin and bovine serum albumin was 44.065%. Blastp analysis revealed six similar sequences between α-livetin and bovine serum albumin, shown in Table 2, which were residues 26–35 in the α-livetin amino acid sequence and residues 24–32 in the serum albumin amino acid sequence. These were residues 80–94 in the α-livetin amino acid sequence and residues 312–326 in the serum albumin amino acid sequence. These were residues 115–125 in the α-livetin amino acid sequence and residues 112–122 in the serum albumin amino acid sequence. These residues occupied 323–335 position in the albumin sequence and residues 525–554 in the serum albumin amino acid sequence. The residues 525–535 in the α-livetin amino acid sequence and residues 520–530 in the serum albumin amino acid sequence were observed. These residues were 560–570 in the α-livetin amino acid sequence and residues 555–565 in the serum albumin amino acid sequence. In addition, there are relatively few studies on cross-reactivity allergy between eggs and other foods at home and outside, so conducting studies on egg cross-reactive allergens is of great significance.

**Table 2:** Similar sequences of α-livetin and bovine milk serum albumin.

α-livetin	Bovine milk serum albumin	Score	E-value	Percent identities	High similarity zone	Vacancy
R_26_DAEHKSEIA_35_	R_24_DTHKSEIA_32_	24.0	4e^−5^	80%	80%	10%
C_80_VANEDAPECSKPLP_94_	C_312_IAEVEKDAIPENLP_326_	12.1	1.6	50%	55%	33%
A_115_DCCSKADPER_125_	A_112_DCCEKQEPER_122_	28.2	1e^−6^	73%	81%	0%
F_323_DEKPADLPSLVE_335_	F_525_DEKLFTFHADICTLPDTEKQIKKQTALVE_554_	18.0	0.013	37%	36%	56%
Y_525_VPPPFNPDMF_535_	Y_520_VPKAFDEKLF_530_	15.5	0.1	45%	63%	0%
L_560_IKRKPQMTEE_570_	L_555_LKHKPKATEE_565_	20.6	7e^−4^	64%	63%	0%

## 6. Effects of processing methods on egg allergens

Hypoallergenicity is defined as a decrease in the ability to cross-link IgE molecules within the body and induce allergic reactions. The development of hypoallergenic egg products can be achieved through processing methods. Food processing can reduce the allergenicity of food proteins by disrupting or shielding specific epitopes and altering the digestibility of these proteins. However, there are also instances where primitive hidden epitopes emerge or new epitopes are formed, increasing the food’s allergenicity (62, 63). Protein allergenicity varies depending on the protein, matrix, processing method, application parameters, and individual sensitivity of each patient. A food product may exhibit different allergenicity in various processing environments. Therefore, investigating the available food processing methods that can reduce the IgE binding capacity and induce *in vivo* tolerance response-ability for egg allergy is of considerable significance. Hypoallergenic egg products have been reported to be unresponsive to a significant proportion of egg-allergic patients (64).

Common processing methods for eggs in daily life include microwaving, steaming, and baking, in addition to radiation exposure, enzymatic hydrolysis, glycosylation, high hydrostatic pressure application, and other methods. Regarding the effect of processing methods on egg white allergens, mainly ovalbumin and OVT have been studied. The IgE binding capacity of ovalbumin can be reduced by heating, radiation exposure, enzymatic hydrolysis, glycosylation, and polyphenol oxidase-mediated cross-linking, leading to conformational changes or overlap of reactive sites with epitopes. Further, heating and radiation exposure can also reduce the allergenicity of ovalbumin *in vivo*. In another study, Tong et al. (65) heated OVT from 55°C to 100°C and showed through an indirect enzyme-linked immunosorbent assay (ELISA) that the potential allergenicity of OVT is closely related to changes in its structure. They also found that the potential allergenicity of OVT heated at a lower temperature increased as its conformation unfolded. On the other hand, heating the protein at above 80°C broke its disulfide bonds and reduced the IgE binding capacity of OVT. Shin, Han, and others (66) found that the IgE binding capacity of OVT almost disappeared when boiled for 30 min, fried for 3 min (frying temperature unknown), or baked at 170°C for 20 min, indicating that the allergenicity of this protein is susceptible to high temperature.

Regarding egg yolk, patients with bird-egg syndrome exhibit allergic symptoms upon ingesting raw egg yolk rather than hard-boiled egg yolk, indicating that egg yolk allergens are heat-labile (67). These findings are further corroborated by reports stating that most egg-allergic patients can safely consume heated egg yolk (68). Another study found an 88% reduction in IgE reactivity of α-livetin after eggs were heated at 90°C for 30 min (69). Reports on food processing suggest that vitellomucoid plays a limited role in reducing egg allergenicity. Although more than half of egg-allergic patients can tolerate boiled eggs, vitellomucoid seems resistant to heating and glycosylation. Uncontrolled processing parameters may even increase the allergenicity of this protein. It is evident that only a combination of several factors, such as processing parameters, matrix, and physicochemical properties of egg allergens, can help control egg allergenicity.

Currently, research on the effects of different processing methods on egg allergens mainly focuses on egg white, while more in-depth exploration is needed for food processing methods that can reduce the IgE binding capacity of egg yolk allergens and induce immune tolerance *in vivo*.

## 7. Recommendations and prospects

### 7.1. Strengthen the research on the molecular structural properties of Gal d 5

Both egg white and egg yolk contain multiple allergens. Compared to egg yolk allergens, the role of egg white allergens in food allergy has been studied more extensively. Egg yolk is a more complex system than egg white and contains a considerable proportion of egg lipids. The high levels of lipids make it very challenging to identify egg yolk allergens, resulting in fewer studies. Establishing a high-throughput and efficient separation and purification technology for Gal d 5 is a key step in studying its molecular properties. Obtaining high-purity Gal d 5 enables us to more clearly and accurately analyze the correlation between its structure and allergenicity. Studies on the epitopes of Gal d 5 are mainly based on bioinformatics-based prediction methods, which still need to be further verified through serological techniques. The effect of non-processing methods on the allergenicity of Gal d 5 and its epitopes is a key direction for future research.

### 7.2. Identify and verify cross-reactive allergens of Gal d 5, and targeted analyze the cross-reactive epitope structure

The results of immunoassays have shown that most egg white-allergic patients have specific IgE antibodies against egg yolk proteins in their blood. The co-reactivity to egg white and egg yolk may be attributed to enhanced independent sensitization to cross-reactive proteins or allergens. In this case, both types of allergens are responsible for the pathogenesis and pathophysiology of egg allergy. Among egg yolk allergens, α-livetin has received more attention because it can easily cause severe allergic diseases, such as “bird-egg” syndrome. Identifying and characterizing cross-reactive allergens of Gal d 5, as well as targeting and analyzing the cross-reactive epitopes, hold great significance for the diagnosis and treatment of clinical egg allergy. Establishing a database containing cross-reactive egg allergens and their epitopes can provide a theoretical basis for devising a rational diet for egg-allergic patients to avoid cross-reactivity and safeguard their health.

### 7.3. Developing precise methods for assessing the allergenicity of Gal d 5

There is an increasing demand for precise methods to detect food allergens. Allergens often exist at trace levels, necessitating detection methods for allergenic components in food with high specificity and sensitivity to track minute quantities of target allergens in complex food matrices (including processed foods) and prevent health risks caused by cross-reactive allergens in food. *In vitro* methods for detecting food allergens primarily rely on immunochemistry, DNA, and mass spectrometry techniques. Currently, no detection methods for Gal d 5 have been reported yet. Analyzing the Gal d 5 antigen-specific epitopes and thus establishing high-specificity antibodies based on these epitopes are important conditions for developing precise immunoassays for this antigen. Mass spectrometry-based peptide “fingerprints” can overcome the effects of allergen cross-reactivity in immunoassays and the disadvantage of DNA-based techniques that cannot directly detect allergenic proteins. Notably, there are many difficulties in establishing efficient animal models for food allergies, such as the severity of allergies, sensitization routes, the dosage of allergens, types and amounts of adjuvants used, and other conditions that need to be optimized. Animal models for evaluating the mechanism underlying Gal d 5 allergy are still in the early stages.

## Author contributions

WJ is responsible for article retrieval and writing. CY is responsible for the arrangement and revision of the content of the article and the writing of the outlook of the article. All authors contributed to the article and approved the submitted version.

## Funding

This work was supported by National Science and Technology Major Project of China (Beijing, 2019YFC1605002) and National Natural Science Foundation of China (Beijing, 31872886).

## Conflict of interest

The authors declare that the research was conducted in the absence of any commercial or financial relationships that could be construed as a potential conflict of interest.

## Publisher’s note

All claims expressed in this article are solely those of the authors and do not necessarily represent those of their affiliated organizations, or those of the publisher, the editors and the reviewers. Any product that may be evaluated in this article, or claim that may be made by its manufacturer, is not guaranteed or endorsed by the publisher.

## Footnotes

^1^ http://www.cbs.dtu.dk/services/TMHMM/
